# PGRMC1 Is a Novel Potential Tumor Biomarker of Human Renal Cell Carcinoma Based on Quantitative Proteomic and Integrative Biological Assessments

**DOI:** 10.1371/journal.pone.0170453

**Published:** 2017-01-20

**Authors:** Dan Zhang, Xiangying Xia, Xixi Wang, Peng Zhang, Weiliang Lu, Yamei Yu, Shi Deng, Hanshuo Yang, Hongxia Zhu, Ningzhi Xu, Shufang Liang

**Affiliations:** 1 State Key Laboratory of Biotherapy and Cancer Center, West China Hospital, Sichuan University, and Collaborative Innovation Center for Biotherapy, Chengdu, P. R. China; 2 Department of Urinary Surgery, West China Hospital, West China Medical School, Sichuan University, Chengdu, P. R. China; 3 Laboratory of Cell and Molecular Biology & State Key Laboratory of Molecular Oncology, Cancer Institute & Cancer Hospital, Chinese Academy of Medical Sciences, Beijing, P. R. China; University of Hong Kong, HONG KONG

## Abstract

Progesterone receptor membrane component 1 (PGRMC1) is widely observed with an elevated level in multiple human cancers. However, the roles of PGRMC1 in renal cancer are not clear and merit further study. In this report, we made a systematic, integrative biological assessment for PGRMC1 in renal cell carcinoma (RCC) by a quantitative proteomic identification, immunohistochemical detection, and its clinic pathologic significance analysis. We found that PGRMC1 abundance is increased by 3.91-fold in RCC tissues compared with its autologous para-cancerous tissues by a quantitative proteome identification. To validate the proteomic result with more confidence, 135 clinic RCC tissues were recruited to measure PGRMC1 abundance by immunohistochemical staining, and 63.7% RCC samples (n = 86) showed a higher abundance of PGRMC1 than the noncancerous counterparts. And the elevated PGRMC1 level was related to the tumor malignancy degree and overall survival of RCC patients. Meanwhile the average serum PGRMC1 concentration for RCC patients (n = 18) was significantly increased by 1.67 fold compared with healthy persons. Moreover an exogenous elevated abundance of PGRMC1 by plasmid transfections significantly enhanced cell proliferation of renal cancer cells *in vitro*. Our findings demonstrate PGRMC1, which promotes RCC progression phenotypes in vitro and in vivo, is a novel potential biomarker and therapeutic target for RCC.

## Introduction

Renal cell carcinoma (RCC), which originates from the proximal tubular epithelium, is the most lethal genitourinary cancer proximately with 209,000 new cases and 102,000 deaths per year worldwide [1], accounting for about 3% of all adult malignancies. RCC is often asymptotic and about 30% of patients diagnosed at the metastatic stage because of the dismal prospects for detection [2]. Biomarkers for early diagnosis of RCC are rare. So it is important to find useful RCC marker candidates to confirm the clinic identification, no matter benign or malignant.

Many novel high-throughput approaches have been adopted to search possible novel biomarkers and therapeutic targets for RCC [3–5]. One of which is proteomics that has offered great promise in profiling potential biomarkers within a large scale and exploring molecular mechanisms in tumorigenesis. The stable isotope labeling by amino acids in cell culture (SILAC) combined with mass spectrometry (MS) is a metabolic labeling strategy for quantitative proteomic analysis, which has gained great advantages, including the ease of implementation, high quality of quantitative data and sensitivity for potential biomarker identification [5]. Based on our previous work in SILAC-based proteomic approach to identify the amount of 14-3-3 isoforms in human RCC tissues [6, 7], we further investigated the differential proteome between human RCC and noncancerous kidney tissues by SILAC-MS dissection, by which PGRMC1 was identified a new differential protein and further confirmed its roles in human RCC.

PGRMC1 (progesterone receptor membrane component 1), with a large cytochrome b5/heme-binding domain, belongs to the membrane-associated progesterone receptor protein family [8]. Recent studies have implicated that PGRMC1 plays important roles in multiple cancers [8, 9], including breast cancer [10, 11], ovarian cancer [12] and lung cancer [13, 14]. Furthermore, PGRMC1 regulates susceptibility of cancer cells to chemotherapy [12, 14, 15]. Due to its correlation with tumor malignancy and progression, PGRMC1 becomes an attractive target of therapeutic intervention for cancer treatments [16]. However, the biological roles of PGRMC1 in RCC are not very clear, and additional researches are merited.

In order to gain a better understanding of the significance of PRGMC1 in renal cell carcinogenesis, we explored a quantitative proteome profiling and clinic pathologic association significance of biopsy samples that obtained from patients with RCC. Our results suggest that PGRMC1 is a novel biomarker and therapeutic target for RCC since its up-regulation promotes renal cancer cell proliferation and predicts tumor malignancy *in vivo*. Additionally we provide a method to detect serum concentration of PGRMC1 secretion as an indicator in clinical diagnosis and therapy prognosis for RCC patients in the foreseeable future.

## Materials and Methods

### RCC tissues and serum samples

A prior review and consent for this project were approved by the Institutional Ethics Committee of State Key Laboratory of Biotherapy, West China Hospital, Sichuan University. The informed written consents on the study were obtained from the RCC patients. 135 pairs of human renal carcinoma tissues (RCTs) and autologous para-cancerous kidney tissues (PKTs) were surgically resected to collect in West China Hospital, Sichuan University (Chengdu, P. R. China). And approximately 5 ml blood was respectively collected pre-nephrectomy from 18 RCC patients and 12 healthy persons with their informed consent. The RCC patients’ clinical information, including the patient age, gender, and histological type of tumor differentiation [17, 18], was also collected with an informed consent.

### Serum sample preparation

Being clotted for 2h at room temperature, the supernatant serum from 5ml blood was collected by centrifugation at 3000 rpm for 15min [19]. 100μL serum was used for ELISA analysis to compare PGRMC1 concentration between RCC patients and healthy persons.

To eliminate the separation interference of serum high-abundant proteins on SDS-PAGE, the high-abundant serum albumin and IgG proteins were deleted before Western blotting analysis on serum PGRMC1. In brief, 40μL serum was processed to remove high- abundant proteins using a commercial removal reagent (ProteoExtract™ Albumin/IgG Removal Kit, Cat. No. 122642, Calbiochem, San Diego, CA) according to user protocols. The main high-abundant proteins, including albumin and IgG proteins, were efficiently deleted with this kit, which had been reported in our previous paper [19]. The pretreated serum sample was precipitated by adding 4 × volume of ice-cold acetone at -20℃ overnight. Then the deposit was dissolved in 200μL RIPA buffer, from which 20μg pretreated serum proteins were run SDS-PAGE to compare serum PGRMC1 concentration between RCC patients and healthy persons by Western blotting. The total loading pretreated serum proteins were visualized by Ponceau-S staining to take as a comparison control.

### Cell culture and SILAC labeling

The deuterated-leucine (Leu-d_3_) labeling HEK293 cells were cultured in DMEM medium with 10% dialyzed fetal bovine serum (FBS), in which Leu-d_3_ (Cambridge Isotope Laboratories, UK) was supplemented to replace normal L-Leu (Leu-d_0_) (GIBCO) [6, 7]. The renal cancer cells OS-RC-2 and 786–0 were cultured in RPMI-1640 media.

### Protein extraction

To avoid heterogenicity of individual tumor tissue, we mixed 3 RCC tissues to extract tumor proteins for following MS identification. Simultaneously, 3 corresponding PKTs were blended together to extract proteins as the noncancerous protein counterparts. The frozen tissues were grinded into powder, and lysed with RIPA buffer (50mM Tris-HCl (pH 8.0), 150mM NaCl, 0.1% SDS, 1.0% NP-40, 0.5% deoxycholate and protease inhibitor cocktail 8340 (Sigma, St. Louis, MO, USA)) to be individually homogenized. As for labeling HEK293T cells, 1×10^6^ cell pellets were dissolved with 1mL RIAP buffer to extract cellular proteins. Tissue or cell extraction was centrifuged at 15 000 rpm for 30 min at 4℃, and the supernatant was collected [7]. Protein concentrations were respectively quantified usually in the range of 6–12 mg/ml using the Protein Assay Kit (500–0006, Bio-Rad, Hercules, CA, USA).

### MS/MS quantification

Same amount of proteins from the labeling HEK293 cells were respectively mixed equally with tissue proteins from RCTs and PKTs, then the protein samples were separated on a 12% SDS-PAGE and Coomassie stained to visualize gel bands. The gels were excised and subjected to in-gel digestion overnight using MS-grade trypsin (Promega, Madison, WI, U.S.A.). The peptides were extracted and subject to MS/MS analysis [7, 19].

Two independent MS/MS experiments were performed for each sample. Proteins were identified by LC-nanospray-MS/MS analysis using a QSTAR XL mass spectrometer (Applied Biosystems, USA). The Mascot (version 1.0) was used as the database search engine for peptide matching and protein identifications against the Swiss-Prot human database, with a false discovery rate of 1%. Protein isoforms and proteins that cannot be distinguished based on the identified peptides are grouped. Only proteins that were identified in both biological samples were included in further analysis. The main parameters for database searching were set as following: (i) the mass tolerance was set as 0.3 Da for MS and MS/MS; (ii) trypsin enzyme specificity and two max-missed cleavages were allowed; (iii) variable modifications including oxidation of methionine and Leu-d_3_ labeling. The SILAC ratio of a peptide in the protein sample was defined as the isotope peak intensity ratio of the unlabeling tissue proteins versus the Leu-d_3_-labeling cellular proteins from HEK293 cells [6, 7]. At least one Leu-containing peptide was used to quantify protein in SILAC-MS analysis, and the protein concentration was averaged when several peptides were taken to quantify a given protein. The relative protein level was quantified by tracking pairs of labeling and unlabeling peptides from the MS spectra [6, 7, 20].

### Immunohistochemistry

Tissue samples were paraffin-embedded to cut into sections with 4μm thickness for hematoxylin-eosin (HE) and immunohistochemistry (IHC) analysis mainly according to our previous protocols [21]. The primary anti-PGRMC1 antibody (ab48012, Abcam) was used at a dilution of 1:200. The second antibody was a biotinylated anti-goat IgG (ZB-2306, ZSGB-BIO Corp., Beijing, China). Tissue slices were visualized by 3, 3-diaminobenzidine solution and counterstained with hematoxylin. Meanwhile the primary antibody was substituted by phosphate-buffered saline (PBS) serving as a control sample.

An estimated percentage of IHC staining was determined by calculating the average number of positive stained cells from 3–4 microscopic fields under 400-fold magnification. The score for positive staining cells was defined as 0–4 with the percent of positive cells separately ranged from 0–5%, 6–25%, 26–50%, 51–75% and over 75%. Similarly the staining intensity was divided into 4 levels: 0, negative; 1, weak; 2, moderate; 3, strong. The final immunoreactivity score for each tissue slice was defined as the staining intensity multiplied by percentage of positive cells [22]. The scores, ranging from 0 to 12, indicate different PGRMC1 abundance in tissues, including a negative (0, -); weak (1–3, +), moderate (4–7, ++) and strong PGRMC1 abundance (8–12, +++).

### ELISA

The PGRMC1 concentration in human serum was measured by the ELISA (CUSABIO, CSB-EL017876HU). Serum samples were added into a 96-well plate with pre-coated anti-PGRMC1 antibody (ab48012, Abcam) to incubate for 1h at 37°C, then the plate was washed with PBST buffer (3.2 mM Na_2_HPO_4_, 0.5 mM KH_2_PO_4_, 1.3 mM KCl, 135 mM NaCl, 0.05% Tween 20, pH 7.4) for 5 times, and incubated with the secondary antibodies for 1h at 37°C. Being stopped by adding 1 N HCl, the absorbance in the 96-well plate was detected at 450 nm on an ELISA Reader (Multiskan Mk3, thermo) with the correction factor at 570 nm.

### Western blot

To validate the accuracy of MS quantification for PGRMC1, PGRMC1 concentration in tissue or serum sample was also detected by Western blotting. 40ug tissue proteins, or 20μg serum samples without high-abundant proteins were separated on a 12% SDS-PAGE gel, then transferred onto a PVDF membrane. Subsequently the membrane was incubated in TBST buffer (20mM Tris-HCl, pH 7.6, 150mM NaCl, 0.1% Tween-20) with 5% non-fat milk to block nonspecific binding at room temperature for 1 h. Then the PVDF membrane was incubated with the anti-PGRMC1 antibody (ab48012, Abcam) at a dilution of 1:1000 in TBST with 1% non-fat dry milk overnight at 4°C. The PVDF membrane was washed with the blocking solution and incubated with the HRP-conjugated secondary antibody with a dilution of 1:10000 (ZhongShan-Golden Bridge, China) at 37°C for 1h. Detection was carried out using the ECL reagent (Amersham Biosciences, Piscataway, New Jersey, USA). For the cellular protein sample, β-actin was taken as a comparison control for western blotting.

### Cell viability assay

The mammalian expressing vector pMIR-DFT with double Flag sequences [23], was used to express exogenous PGRMC1 in transiently transfected cells. The PGRMC1 gene was cloned into the *Bam* HI and *Xho* I sites of the vector. 5×10^3^ OS-RC-2 cells per well were seeded into a 96-well plate for transfection of the recombinant plasmid Flag-tagged PGRMC1 (pFlag-PGRMC1) by polyethyleneimine (PEI) [24] at 1:1 weight ratio for 24-96h and measure cells’ growth. Each time point was performed in parallel with 3 repetitions.

Meanwhile, the shRNA plasmid against PGRMC1, which ordered from Santa Cruz Biotechnology (sc-76111-SH, Santa Cruz Biotechnology, CA), was transfected to 5×10^3^ OS-RC-2 cells in a 96-well plate to monitor cell proliferation.

Cells were cultured in RPMI 1640 (Gibco, Gaithersburg, MD) (L-glutamine and 25mM HEPES) media with 10% FBS, 100μg/mL streptomycin (Life Technologies, Grand Island, NY). Cell viability was measured by the methylthiazoletetrazolium (MTT) method and with 3 biological repeats, the final result was statistically estimated. All data were presented as the mean ±Standard Deviation (SD). Cell viability was mathematically present as absorption in test divided by the control.

## Results and Discussion

### Bioinformatic analysis on differential proteins

In MS experiments, 1099 proteins were both identified in RCTs and PKTs. And 931 proteins were successfully quantified, 97% of which were determined by the isotopic intensity ratios of two or multiple Leu-containg peptides, only 3% being quantified by one. The average SD, calculated by the isotopic intensity ratios from multiple Leu-containg peptides, was 0.17 for all 931 quantified proteins. The change ratio from 1.3 to 2.0 is often used as cut-off value for both statistical and biological significance [7, 20]. Here we defined the altered protein with its change ratio above 1.34 or below 0.66 as a significantly up-regulated or down-regulated one between RCTs and PKTs. Totally 82 proteins, including 69 up-regulated and 13 down-regulated ones, were detected (S1 Table). From which, more than 20 proteins have been identified by other research groups before, such as PKM2, an nexin family proteins, vimentin, heat shock proteins [25–27] and tyrosine 3-monooxygenas [28].

**Fig 1:**
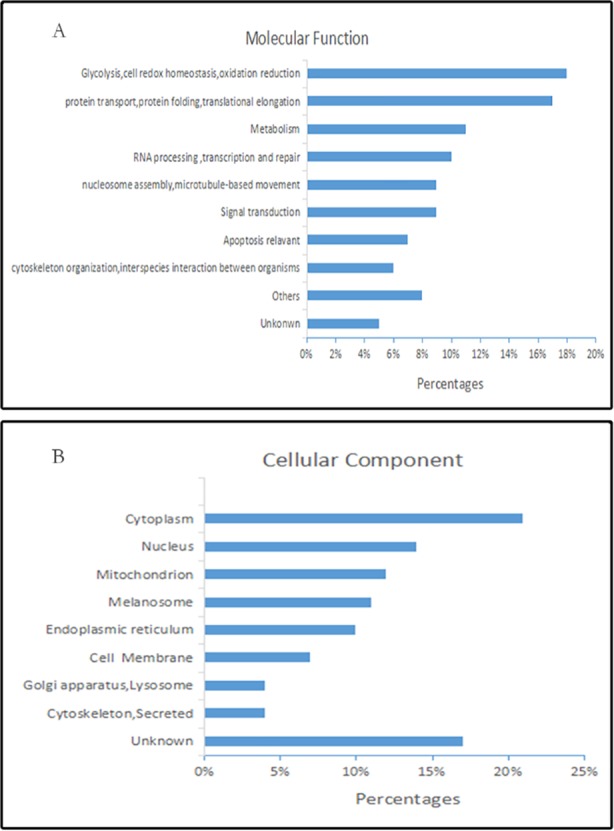
Bioinformatic analysis of 82 differential proteins in RCC. GO enrichment analysis of differential proteins based on molecular function **(A)** and cellular component **(B).**

Based on the classification of Gene Ontology (GO) annotation (http://www.ncbi.nlm.nih.gov/), 82 changed proteins are involved in multiple cell functions (Fig 1). Among these proteins, 18.2% of which, including pyruvate kinase isozyme M2, fructose-bisphosphate aldolase A, isoform 1 of pyruvate dehydrogenase E1 component subunit beta and peroxiredoxin-6, are involved in glycolysis [25, 27], cell redox homeostasis and oxidation reduction[26]. And 17.1% play functions in protein transport, protein folding and translational elongation such as HSP90B1, PPIB, and CANX. In addition, 8 proteins take part in signal transduction, as well as 9 influencing on cell metabolism. The cellular distributions are very wide, mainly locating in cytoplasm (21%), nucleus (14%), cell membrane (7%), mitochondrion (12%), endoplasmic reticulum (10%) and other cell fractions (Fig 1B).

### An up-regulated protein PGRMC1 was identified by MS

In SILAC-based MS analysis, the ‘SILAC ratio 1’ represents the relative abundance of a certain protein between PKTs *versus* HEK293 cells [7]. Similarly, the ‘SILAC ratio 2’ indicates the relative concentration between RCTs *versus* HEK293 cells. Using the Leu-d_3_-labeling cellular proteins as internal standards in MS, the relative protein abundance between two different tissues (RCTs *versus* PKTs), namely change ratio, can be acquired by calculating the ratio of ‘SILAC ratio2’ *versus* ‘SILAC ratio 1’ (SILAC ratio2 / SILAC ratio1). We had applied the SILAC-MS method to respectively distinguish the levels of 14-3-3 isoforms in renal cancer and glioma before [6, 7, 20].

**Fig 2:**
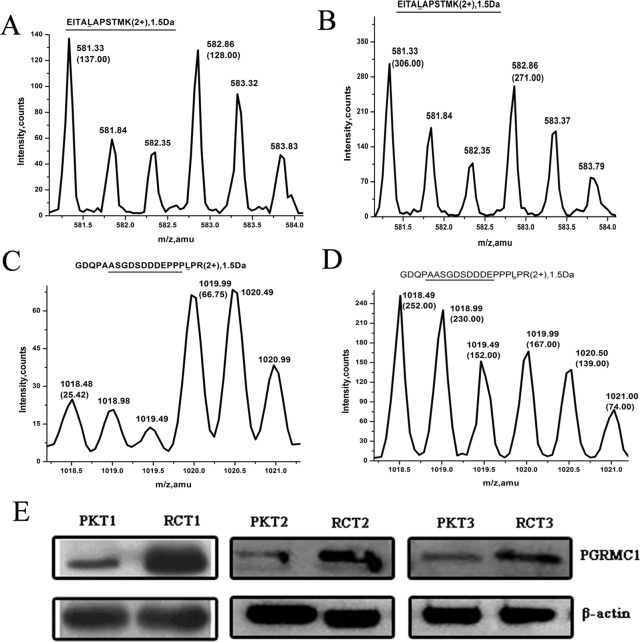
The relative abundance of PGRMC1 compared between RCTs and PKTs. The SILAC ratios of β-actin with isotope labeling peptides “EITALAPSTMK” (m/z 582.86/581.33, 2+) respectively from the group of protein mixture composed of PKTs and HEK293 cells **(A)**, another group of protein sample containing RCTs and HEK293 cells **(B)**. The isotope labeling peptides ‘GDQPAASGDSDDDEPPPLPR’ (m/z 1019.99/1018.49, 2+) from protein mixture containing PKTs and HEK293 cells **(C)**, RCTs and HEK293 cells **(D)** were used to quantify PGRMC1 abundance. PGRMC1 concentration was validated between 3 RCTs and corresponding PKTs by western blotting **(E)**. RCTs: renal cell carcinoma tissues; PKTs: para-cancerous kidney tissues.

To normalize the internal standard for MS quantification, we detected the change ratio of β-actin based on the isotope labeling Leu-containing peptide (EITALAPSTMK) between RCTs and PKTs (Table 1 & Fig 2). In the first time of MS/MS identification (Table 1, Exp1), the SILAC ratio 1 was 1.07 (137 counts/128 counts, Fig 2A). And the SILAC ratio 2 was 1.13 (306 counts/271 counts, Fig 2B). So the change fold of β-actin, calculated from SILAC ratio 2 *versus* SILAC ratio 1, was 1.06, indicating a nearly equal concentration of β-actin between RCTs and PKTs. And the change fold 1.01 of β-actin in the second MS/MS quantification (Table 1, Exp2) was well consistent with the above data. So an equal abundance of β-actin between RCTs and PKTs suggest that the same loading sample amount was ensured to be a comparison base for other proteins in MS analysis.

**Table 1:** Quantitative MS data of PGRMC1 in renal carcinoma.

Accession^a^	Protein	Exp	Score^b^		Peptide^c^		SILAC-ratio^d^		Change ratio	Average
			RCTs	PKTs	RCTs	PKTs	ratio2	ratio1		
O00264	PGRMC1	Exp1	312(10)	160(7)	2	1	1.51±0.12	0.38	3.98	3.91±0.10
		Exp2	312(10)	110(7)	2	2	1.96±0.43	0.51±0.30	3.84	
P60709	β-actin	Exp1	312(19)	328(16)	5	5	1.01±0.09	0.95±0.08	1.06	1.03±0.03
		Exp2	312(19)	255(18)	5	5	0.98±0.07	0.97±0.03	1.01	

^a.^ UniProtKB number

^b^ MOWSE scores (Queries match)

^c.^ Leu- containing peptide

^d^ Mean±SD

We specially noticed the protein PGRMC1 was increased to 3.91-fold in RCTs compared with PKTs based on the SILAC-MS detection (Table 1), and its up-regulation has not been previously reported in RCC by now. As an example, one isotope peptide (GDQPAASGDSDDDEPPPLPR) of PGRMC1 was displayed its abundance comparison between RCTs and PKTs in detail (Fig 2). In one MS/MS quantification (Table 1, Exp1), the SILAC ratio1 0.38 was calculated from the peak intensity ratio of the pair of isotope peptides (m/z1018.49 versus m/z1019.99, 25.42 counts/66.75counts) (Fig 2C). Similarly, the SILAC ratio 2 was 1.51 (252 counts/167 counts) (Fig 2D). So the final change ratio of PGRMC1 between RCTs and PKTs (SILAC ratio2/ SILAC ratio1) was 3.98. And a same data processing way in another time of MS identification (Table 1, Exp2) was shown a similar 3.84-fold abundance for PGRMC1 in RCTs. Therefore, the average change fold of PGRMC1 was 3.91, which indicates that PGRMC1 abundance was greatly elevated in RCC tissues than the noncancerous counterparts. Furthermore, three pairs of randomly chosen RCTs and PKTs were also verified with an elevated PGRMC1 concentration in RCC by Western blotting detection (Fig 2E), which was consistent with the MS data.

The MS proteomics data have been deposited to the ProteomeXchange Consortium via the PRIDE [29] partner repository with the dataset identifier PXD004595 (S1 File).

PGRMC1 has been widely found to be an elevated concentration in multiple human cancers [10–14], which means the abnormal level of this protein is possibly related with carcinogenesis. However, the associations of PGRMC1 with renal cancer are not clear. So we focused on the relationship of PGRMC1 up-regulation with RCC in the following investigation.

### PGRMC1 is confirmed to widely increase in RCC tissues

**Fig 3:**
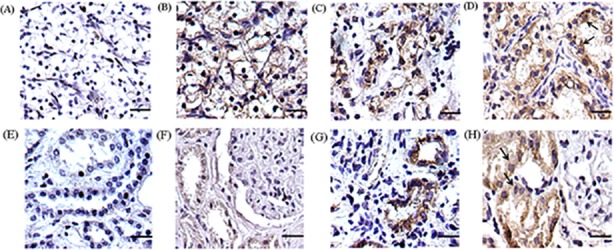
**Different staining level and protein distribution of PGRMC1 in RCTs (A-D) and PKTs (E-H)**. A negative, weak, moderate and strong staining pattern of PGRMC1 was respectively shown in RCTs **(A-D)** and PKTs **(E-H)**. PGRMC1 mainly located in cytoplasm and cell membrane in RCTs as the arrow indicated **(D)**. PGRMC1 was observed in the cytoplasm of the convoluted tubules in PKTs **(H)**. The scale bar represented 100 μm (original magnification×400).

To further analyze PGRMC1 abundance on a large scale of RCC biopsy tissues, 135 pairs of RCTs and PKTs were applied to compare the protein abundance by IHC. Except for one negative tissue, 134 RCTs (99.3%) had a PGRMC1-positive immunoreactivity (Table 2). Among 135 RCTs, 38 cases (28.1%) showed a strong abundance with scores 10.57±1.38 (Table 2 & Fig 3D), and 48 cases (35.6%) showed a moderate level with scores 7.00±1.01 (Fig 3C), while other 48 cases (35.6%) had a weak staining level with scores 2.77±1.16 (Fig 3B). Moreover cell distribution of PGRMC1 in RCTs was located in cytoplasm and cell membrane (Fig 3D).

**Table 2:** PGRMC1 immunoreactivity between RCTs and PKTs.

Immuno -reactivity	RCTs (n = 135)		PKTs (n = 135)	
	Percentage	Average score	Percentage	Average score
−	0.7%(1/135)	0	2.2%(3/135)	0
+	35.6%(48/135)	2.77±1.16	67.4%(91/135)	2.86±0.96
++	35.6%(48/135)	7.00±1.01	22.2%(30/135)	6.13±0.78
+++	28.1%(38/135)	10.57±1.38	8.2%(11/135)	9

−: Negative; +: weak immunoreactivity; ++: moderate immunoreactivity

+++: strong immunoreactivity.

On the other hand, 132 cases of PKTs (97.8%) showed a positive staining of PGRMC1, including 91 cases (67.4%) with a weak level (Fig 3F), 30 cases (22.2%) with moderate abundance (Fig 3G), and 11 cases (8.2%) with a strong PGRMC1 level (Fig 3H). In PKTs, PGRMC1 was predominately in cytoplasm of the convoluted tubules (Fig 3H). In conclusion, RCTs displayed a significantly higher abundance of PGRMC1 than PKTs (p<0.01) (Table 2).

### PGRMC1 up-regulation relates with RCC malignancy and patient’s poor survival

Furthermore, combining with the IHC data and the clinical information of renal cancer samples, we discovered correlations between PGRMC1 abundance and RCC pathologic features. Among the 135 RCC patients with a mean 60-years’ old, 81 cases were male, and 54 were female (Table 3). The abundance of PGRMC1 showed no obvious differences between gender and age (p>0.05). While the PGRMC1 abundance in RCC is statistically correlated with the tumor malignancy degree (TNM stage). In 135 pairs of RCTs, RCC cases with TNM III-IV (n = 40) showed a much higher up-regulation of PGRMC1 than those with TNM I-II (n = 95) (P<0.05, Table 3).

**Table 3:** Correlations between PGRMC1 abundance and clinical characteristics of RCC.

Factors	No. of patients (%)	Staining degree (average scores)	P-value
**Gender**			
** Male**	81(60.0%)	++ (6.95±2.97)	
** Female**	54(40.0%)	++ (5.70±2.99)	0.0842
**Age**			
** <60**	69(51.1%)	++ (5.78±3.05)	
** ≥60**	66(48.9%)	++ (5.72±3.01)	0.9312
**TNM stage**			
** I-II**	95(70.4%)	++ (5.74±3.01)	
** III-IV**	40(29.6%)	+++(8.31±2.94)	0.0189

−: Negative; +: weak immunoreactivity; ++: moderate immunoreactivity

+++: strong immunoreactivity.

**Fig 4:**
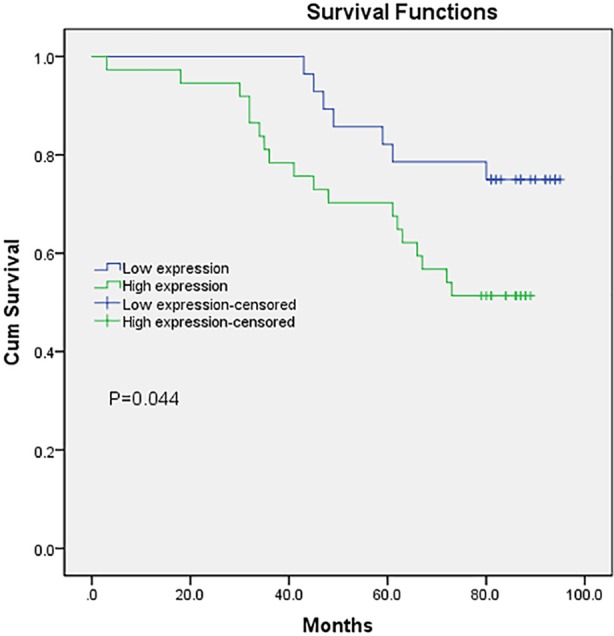
Associations between PGRMC1 abundance and the overall survival period of RCC patients. The difference in overall survival was significant between RCC patients with a low-abundant PGRMC1 (with 1–4 scores) and those with a high-abundant PGRMC1 (>4 scores) (P<0.05).

At the same time, we investigated the relationship between PGRMC1 abundance and RCC patient’s survival time. The kaplan-meier estimate showed 65 RCC patients with a low-abundance of PGRMC1 had a significant longer overall survival time (84.96 ±3.48 months) compared to the patients with a high-abundance of PGRMC (P = 0.044 by the log-rank test, Fig 4), and the mean overall survival for the latter RCC patients was 167.81±4.21 months. In general, the abundance of PGRMC1 in RCC is associated with prognosis, and RCC patients with a high-abundance of PGRMC1 have a poor postoperative overall survival.

### Serum PGRMC1 concentration is increased in RCC patients

**Fig 5:**
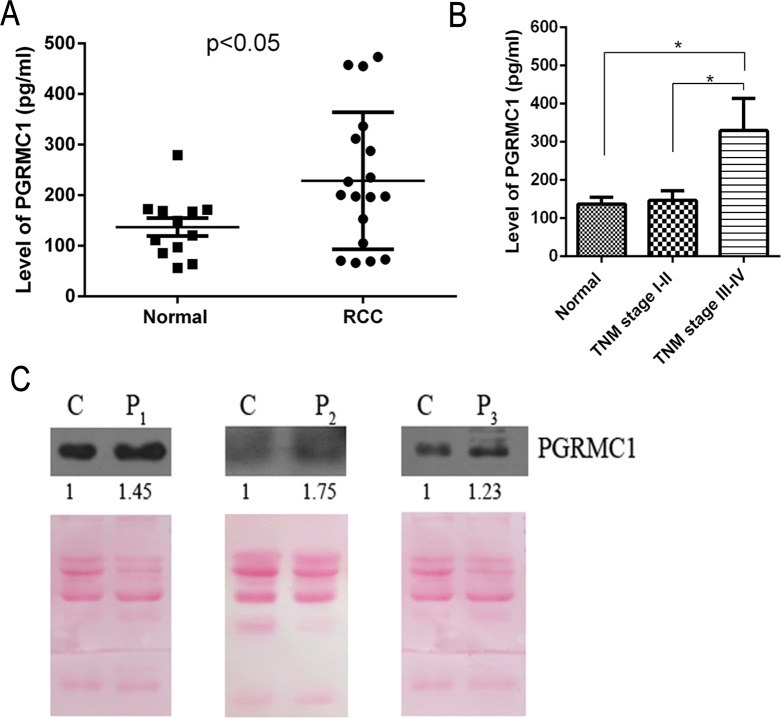
A statistically higher concentration of serum PGRMC1 from RCC patients. The average serum PGRMC1 concentration was higher in 18 RCC patients compared to 12 healthy persons (P<0.05) **(A)**. The serum PGRMC1 concentration was related with the TNM stage of RCC **(B)**. Serum PGRMC1 abundance was detected in three randomly selected RCC patients **(C)**. C: a pool of sera from three healthy persons; P_1_-P_3_: the serum from 3 RCC patients. The total loading pretreated serum proteins were visualized by Ponceau-S staining to take as a comparison control.

Serum biomarker discovery for RCC is active and challenging so far at present [30–31]. In order to know the profiling of serum PGRMC1 concentration, totally 18 cases of serum samples collected from RCC patients were detected by ELISA, and 12 cases of sera from healthy donors were used as normal controls. The average serum PGRMC1 concentration in RCC was 228.6 ± 31.9 pg/mL, while the mean value from healthy persons’ sera was 137.1± 17.8 pg/mL. Actually the mean level of serum PGRMC1 in RCC is significantly increased to almost 1.67-fold compared with healthy persons (P<0.05) (Fig 5A). And the serum PGRMC1 concentration from 3 RCC patients was also verified to increase compared with 3 healthy people by western blotting analysis (Fig 5C).

In order to clarify correlation between disease severity of RCC and serum PGRMC1 concentration, 18 RCC serum samples were further sub-grouped into two types, including 11 RCCs with TNMⅠ-Ⅱ and 7 RCCs with TNM Ⅲ-Ⅳ. The serum PGRMC1 concentration was 329.6±83.88 pg/mL for 7 RCC patients with TNM Ⅲ-Ⅳ, which was statistically higher than the concentration of 146.9±25.37 pg/mL for 7 patients with TNMⅠ-Ⅱ (P<0.05). However, there had no obvious difference for serum PGRMC1 between RCC patients with TNMⅠ-Ⅱ(146.9±25.37 pg/mL) and healthy persons (137.1± 17.8 pg/mL) (P>0.05). In conclusion, serum PGRMC1 concentration is associated with tumor malignancy degree of RCC, and a higher serum PGRMC1 concentration is detected in RCC with TNM Ⅲ-Ⅳ than patients with TNMⅠ-Ⅱand healthy persons.

### Up-regulated PGRMC1 promotes cell proliferation

**Fig 6:**
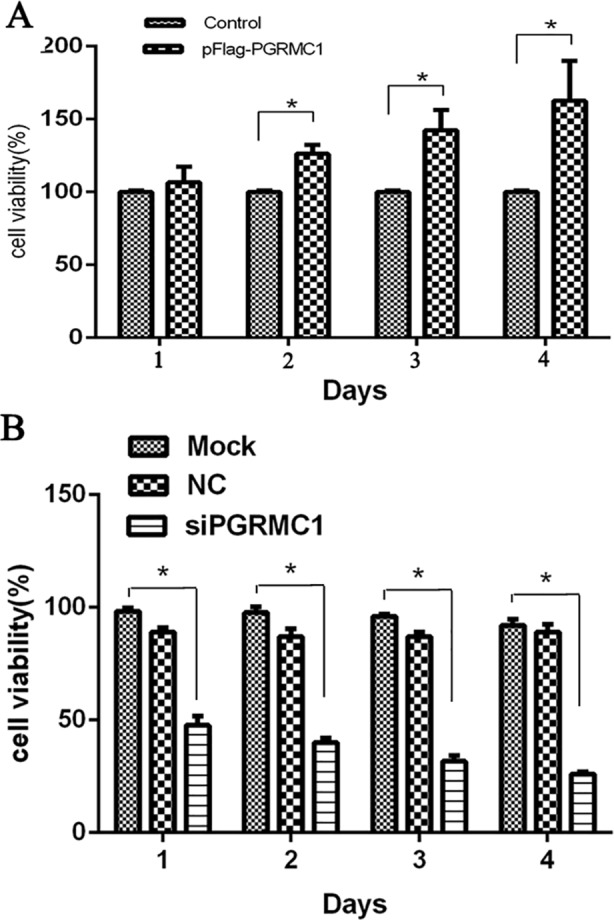
**Cell proliferation under the overexpression (A) or knockdown (B) of PGRMC1 in OS-RC-2 cells.** Each experiment was performed in triplicate. Control: cells transfected with Flag-containing empty plasmids; Mock: cells without treatment; NC: the nonspecific siRNA sequences. * p < 0.05.

Furthermore, we assayed cell growth change in renal cancer cells with transiently overexpression or knockdown of PGRMC1 gene respectively. As results, cell growth, with transiently transfected pFlag-PGRMC1 plasmids in OS-RC cells for 48-96h, was at least increased to 155% (Fig 6A), compared with the vehicle controls in which cell number was taken as a baseline (100%) (p<0.05). And on the contrary, the shRNA-mediated knockdown of endogenous PGRMC1 caused a significant decrease to about 50% in cell number compared to either the mock or the non-targeting control siRNA (NC) treated cells (p<0.05) (Fig 6B). Cell growth was meanly detected from three wells in parallel (n = 3), and three independent experiments were performed. Similar results were confirmed in another RCC cell line 786–0 (data not shown). Therefore, the up-regulation of PGRMC1 in renal cancer cells promotes cell proliferation.

## Discussion

Although nearly 300 differential proteins have been identified between renal tumor samples, cell lines or patient’s sera and their respective controls by proteomic studies, RCC-specific biomarkers have not been available for the early detection and predicted responses to therapy so far [26]. The possible reasons are due to the limitation of 2-DE separation of membrane proteins and low-abundant proteins existing. The sensitive SILAC-based quantitative proteomic approach that we used here provides us new opportunities for novel cancer biomarker discovery [4, 5]. By implementing it coupled with the tissue immunohistochemical validation on a large scale, we identified the concentration of PGRMC1 is greatly increased in most of renal cancer tissues when compared with normal kidney tissues.

To our knowledge, it is the first time for us to discover an elevated PGRMC1 widely in renal carcinoma based on a quantitative proteomic analysis for renal carcinoma tissues. Furthermore, we confirmed that PGRMC1 promotes RCC development, and its concentration is related the overall survival of RCC patients. A higher abundance of PGRMC means a worse overall survival for RCC patients. More importantly, PGRMC1 concentration is also significantly increased in sera of RCC patients compared to healthy persons. Because of the tumor heterogeneity and the individual differences, the serum protein concentration frequently has a big difference. For example, it has indicated that the serum PGRMC1 level was significantly different in lung cancer patients with adenocarcinoma, squamous cell lung cancer and large cell lung cancer [32]. So it is possible that the concentration range is much broader and there are quite a number of RCC serum samples with PGRMC1 concentration similar to healthy controls. However the mean serum PGRMC1 concentration in RCC is generally increased to almost 1.67-fold compared with healthy persons (P<0.05) (Fig 5A). Moreover a higher serum PGRMC1 concentration is detected in RCC with TNM Ⅲ-Ⅳ than TNM Ⅰ-Ⅱ and healthy persons (Fig 5B). Our studies show serum PGRMC1 concentration may reflect disease progression states of RCC.

In addition, small chemical molecules have been designed to specifically inhibit PGRMC1 activity [33]. New small molecule drug targeting PGRMC1 is promising to develop for RCC therapy. Because PGRMC1 concentration is tightly associated with RCC development and survival time of patients, so the detection of serum PGRMC1 concentration of RCC patients can help conveniently monitor disease states and cellular drug responses for personalized therapies in the future. As we know, the survival period after surgery varies even if the RCC patients are diagnosed at the same clinical stage. By the aid of the prognostic analysis, it will help clinic doctors determine whether an adjuvant chemotherapy will be necessary to reduce the risk of RCC metastasis.

At present, several novel techniques including the SELDI-TOF-MS provide a simple and sensitive approach to verify serum biomarkers from a variety of biological samples without complex pretreatment prior to MS analysis [34]. Some potential serum biomarkers had been identified in RCC [35–37] via a SELDI-TOF-MS method. Although the number of our serum samples is limited at present, the prognostic or diagnostic value of PGRMC1 can be validated among a large scale of newly enrolled RCC persons by employing clinically practical tools such as ELISA analysis in the future.

Renal cancer arises from the complicated interactions of genes, proteins, metabolites and environmental factors, which cannot be featured only at gene or protein levels. In our proteomic analysis, multiple differential proteins were identified between renal cancer tissues compared to the counterparts, and the proteins involve in glycolysis, cell metabolism, cell signal transduction and so on. Our proteomic data supported previous reports that cell activities including TCA cycle, glycolysis, and pyruvate metabolism [38] are altered in renal cancer. Although we demonstrated that a high abundance of PGRMC1 could promote renal cancer cell growth, it is not clear PGRMC1-mediated molecular signaling pathways in carcinogenesis and cancer progression. It is known PGRMC1 can bind with sigma-2 receptor to regulate cell proliferation [39]. PGRMC1 has been proposed to act as a progesterone receptor or progesterone signaling intermediate in multiple cell types. PGRMC1 also interacts with progestin to mediate proliferative effects in breast cancer cells [40]. But it is unclear how PGRMC1 transduces anti-apoptotic signaling by progesterone. However, the purified PGRMC1 does not bind to progesterone [41]. Recent reviews appear to have reached a consensus that PGRMC1 is not by itself a progesterone receptor.

As well known, activation of the EGFR 2-tyrosine kinase has been linked to increased proliferation, angiogenesis, metastasis, and decreased apoptosis. Actually PGRMC1 can bind to EGFR and stabilizes EGFR at the plasma membrane [14]. Moreover, PGRMC1 increases susceptibility to EGFR inhibitors, likely because it increases EGFR levels at the plasma membrane [14]. In contrast, the exogenous PGRMC1 are sometimes localized to the nucleus in ovarian cells [12], and PGRMC1 is also found at the plasma membrane in ovarian and neuronal cell types [42, 43].

PGRMC1 has been identified to be a secreted protein [44]. The secreted PGRMC1 is a good biomarker for detecting the presence of cancer in a subject and for monitoring cancer progression by assaying its levels in a bodily fluid. For example, measuring the level of serum PGRMC1 is useful in assessing the stage of lung cancer, particularly the stage 1 cancer where the cancer limited to the lung and hasn't spread to the lymph nodes and wherein the tumor is generally smaller [44]. It also can be used to assess cancer status including the presence or absence of disease, the risk of developing disease, the stage of the disease, and the effectiveness of treatment of disease in a lung cancer, a breast cancer, an ovarian cancer, an oral cancer or a head or neck cancer [44]. So far we will pay more attentions to clarify PGRMC1 roles and discover PGRMC1-involved molecular signal pathways in RCC development in further study.

In summary, the up-regulated PGRMC1 is statistically correlated with the tumor malignancy degree of RCC TNM stages. Meanwhile the elevated PGRMC1 is more likely present with a poor differentiation. PGRMC1 is potentially become a biomarker and an attractive target for therapeutic intervention for RCC. Proteomic signatures integrated with the clinic-pathological data are promising to sensitively monitor renal cancer progression and dynamically clinical response to drug therapy for RCC.

## Supporting Information

S1 Table The changed proteins were identified between RCTs and PKTs. (DOC)

S1 File Reviewer account information of ProteomeXchange data (PXD004595). (DOC)
