# Supplementary material for: PGRMC1 Is a Novel Potential Tumor Biomarker of Human Renal Cell Carcinoma Based on Quantitative Proteomic and Integrative Biological Assessments
Source: PLoS One. 2017 Jan 20;12(1):e0170453. doi: 10.1371/journal.pone.0170453 (PMC5249100; doi:10.1371/journal.pone.0170453)
Supplement: S1 Table — (DOC) [file pone.0170453.s001.doc]

Supplementary Table 1. Changed proteins identified between RCTs and PKTs

| **ID** | **Gene** | **Description** | **Location** | **Change ratio (RCTs/PKTs)** |
| --- | --- | --- | --- | --- |
| **69 upregulated proteins** | | | | |
| P14625 | HSP90B1 | heat shock protein 90kDa beta (Grp94), member 1 | Cytoplasm | 1.72 |
| Q09666 | AHNAK | AHNAK nucleoprotein | Nucleus | 1.73 |
| P22314 | UBA1 | ubiquitin-like modifier activating enzyme 1 | Cytoplasm | 1.5 |
| Q14697 | GANAB | glucosidase, alpha; neutral AB | Cytoplasm | 1.61 |
| P13639 | EEF2 | eukaryotic translation elongation factor 2 | Cytoplasm | 1.65 |
| P55786 | NPEPPS | aminopeptidase puromycin sensitive | Cytoplasm | 1.75 |
| P16403 | HIST1H1C | histone cluster 1, H1c | Nucleus | 2.13 |
| P14618 | PKM2 | pyruvate kinase, muscle | Cytoplasm | 1.85 |
| P13797 | PLS3 | plastin 3 (T isoform) | Cytoplasm | 1.76 |
| P61978 | HNRNPK | heterogeneous nuclear ribonucleoprotein K | Nucleus | 1.6 |
| P06733 | ENO1 | enolase 1, (alpha) | Cytoplasm | 1.72 |
| P30101 | PDIA3 | protein disulfide isomerase family A, member 3 | Cytoplasm | 1.99 |
| Q15084 | PDIA6 | protein disulfide isomerase family A, member 6 | Cytoplasm | 2.13 |
| P07237 | P4HB | prolyl 4-hydroxylase, beta polypeptide | Cytoplasm | 1.81 |
| P04350 | TUBB4 | tubulin, beta 4 | Cytoplasm | 1.67 |
| Q8N6N5 | TUBB2C | tubulin, beta 2C | Cytoplasm | 1.56 |
| Q13885 | TUBB2A | tubulin, beta 2A | Cytoplasm | 1.54 |
| Q9BQE3 | TUBA1C | tubulin, alpha 1c | Cytoplasm | 2.04 |
| P00352 | ALDH1A1 | aldehyde dehydrogenase 1 family, member A1 | Cytoplasm | 1.68 |
| P07355 | ANXA2 | annexin A2 | Plasma Membrane | 2.09 |
| P08758 | ANXA5 | annexin A5 | Plasma Membrane | 1.68 |
| P04083 | ANXA1 | annexin A1 | Plasma Membrane | 1.56 |
| A6NMY6 | ANXA2P1 | Similar to Annexin A2 | Plasma Membrane | 2.21 |
| Q99623 | PHB2 | prohibitin 2 | Cytoplasm | 1.87 |
| P35232 | PHB | prohibitin | Nucleus | 1.63 |
| P04075 | ALDOA | aldolase A, fructose-bisphosphate | Cytoplasm | 2.04 |
| P09211 | GSTP1 | glutathione S-transferase pi 1 | Cytoplasm | 5.41 |
| O00264 | PGRMC1 | progesterone receptor membrane component 1 | Plasma Membrane | 3.98 |
| P23396 | RPS3 | ribosomal protein S3 | Cytoplasm | 2.08 |
| P62750 | RPL23A | ribosomal protein L23a | Cytoplasm | 1.57 |
| P62851 | RPS25 | ribosomal protein S25 | Cytoplasm | 1.58 |
| Q07020 | RPL18 | ribosomal protein L18 | Cytoplasm | 3.73 |
| P62249 | RPS16 | ribosomal protein S16 | Cytoplasm | 1.96 |
| P09417 | QDPR | quinoid dihydropteridine reductase | Cytoplasm | 1.54 |
| P69905 | HBA2 | Hemoglobin subunit alpha | Cytoplasm | 1.58 |
| P23284 | PPIB | peptidylprolyl isomerase B (cyclophilin B) | Cytoplasm | 2.24 |
| P62937 | PPIA | peptidylprolyl isomerase A (cyclophilin A) | Cytoplasm | 1.95 |
| P15531 | NME1 | Nucleoside diphosphate kinase A | Cytoplasm | 2.35 |
| P18085 | ARF4 | ADP-ribosylation factor 4 | Cytoplasm | 1.5 |
| P84077 | ARF1 | ADP-ribosylation factor 1 | Cytoplasm | 1.72 |
| P30086 | PEBP1 | phosphatidylethanolamine binding protein 1 | Cytoplasm | 1.51 |
| Q99497 | PARK7 | Parkinson disease (autosomal recessive, early onset) 7 | Nucleus | 1.78 |
| P62805 | HIST4H4 | Histone H4 | Nucleus | 2.06 |
| P05413 | FABP3 | fatty acid binding protein 3 | Cytoplasm | 3.1 |
| P61604 | HSPE1 | heat shock 10kDa protein 1 (chaperonin 10) | Cytoplasm | 1.61 |
| Q969H8 | C19ORF10 | chromosome 19 open reading frame 10 | Extracellular Space | 1.73 |
| P08670 | VIM | vimentin | Cytoplasm | 1.82 |
| Q00610 | CLTC | clathrin, heavy chain (Hc) | Plasma Membrane | 1.41 |
| O75369 | FLNB | filamin B, beta (actin binding protein 278) | Cytoplasm | 1.37 |
| Q9Y4L1 | HYOU1 | hypoxia up-regulated 1 | Cytoplasm | 1.42 |
| P11142 | HSPA8 | heat shock 70kDa protein 8 | Cytoplasm | 1.43 |
| P10809 | HSPD1 | heat shock 60kDa protein 1 (chaperonin) | Cytoplasm | 1.37 |
| P09874 | PARP1 | poly (ADP-ribose) polymerase 1 | Nucleus | 1.49 |
| P78527 | PRKDC | protein kinase, DNA-activated, catalytic polypeptide | Nucleus | 1.36 |
| P62258 | YWHAE | tyrosine 3-monooxygenase | Cytoplasm | 1.4 |
| P27824 | CANX | calnexin | Cytoplasm | 1.39 |
| P02545 | LMNA | lamin A/C | Nucleus | 1.46 |
| P20700 | LMNB1 | lamin B1 | Nucleus | 1.35 |
| P05787 | KRT8 | keratin 8 | Cytoplasm | 1.37 |
| P14136 | GFAP | glial fibrillary acidic protein | Cytoplasm | 1.37 |
| P07437 | TUBB | tubulin, beta | Cytoplasm | 1.49 |
| P49448 | GLUD2 | glutamate dehydrogenase 2 | Cytoplasm | 1.39 |
| P11177 | PDHB | pyruvate dehydrogenase (lipoamide) beta | Cytoplasm | 1.43 |
| P22626 | HNRNPA2B1 | heterogeneous nuclear ribonucleoprotein A2/B1 | Nucleus | 1.42 |
| Q99714 | HSD17B10 | hydroxysteroid (17-beta) dehydrogenase 10 | Cytoplasm | 1.48 |
| P60174 | TPI1 | triosephosphate isomerase 1 | Cytoplasm | 1.48 |
| Q9NS69 | TOMM22 | translocase of outer mitochondrial membrane 22 homolog (yeast) | Cytoplasm | 1.43 |
| P99999 | CYCS | cytochrome c, somatic | Cytoplasm | 1.47 |
| A0A024R9H2 | HRSP12 | heat-responsive protein 12 | Cytoplasm | 1.34 |
| **13 downregulated proteins** | | | | |
| Q07065 | CKAP4 | cytoskeleton-associated protein 4 | Cytoplasm | 0.64 |
| P21281 | ATP6V1B2 | ATPase, H+ transporting, lysosomal 56/58kDa, V1 subunit B2 | Cytoplasm | 0.66 |
| P30041 | PRDX6 | peroxiredoxin 6 | Cytoplasm | 0.65 |
| P01620 | SIE | Ig kappa chain Ⅴ-Ⅲ region SIE | Other | 0.66 |
| O75891 | ALDH1L1 | aldehyde dehydrogenase 1 family, member L1 | Cytoplasm | 0.62 |
| P49419 | ALDH7A1 | aldehyde dehydrogenase 7 family, member A1 | Cytoplasm | 0.56 |
| P05091 | ALDH2 | aldehyde dehydrogenase 2 family (mitochondrial) | Cytoplasm | 0.64 |
| P05023 | ATP1A1 | ATPase, Na+/K+ ransporting, alpha 1 polypeptide | Plasma Membrane | 0.27 |
| Q08426 | EHHADH | enoyl-Coenzyme A,hydratase | Cytoplasm | 0.49 |
| P30084 | ECHS1 | enoyl Coenzyme A hydratase, short chain, 1, mitochondrial | Cytoplasm | 0.58 |
| P01834 | IGKC | immunoglobulin kappa constant | Extracellular Space | 0.6 |
| P35354 | COX2 | cytochrome c oxidase II | Cytoplasm | 0.36 |
| P13073 | COX4I1 | cytochrome c oxidase subunit IV isoform 1 | Cytoplasm | 0.38 |
