# Supplementary material for: PGRMC1 Is a Novel Potential Tumor Biomarker of Human Renal Cell Carcinoma Based on Quantitative Proteomic and Integrative Biological Assessments
Source: PLoS One. 2017 Jan 20;12(1):e0170453. doi: 10.1371/journal.pone.0170453 (PMC5249100; doi:10.1371/journal.pone.0170453)
Supplement: S1 File — (DOC) [file pone.0170453.s002.doc]

Reviewer account:

Username: [reviewer87808@ebi.ac.uk](mailto:reviewer87808@ebi.ac.uk),

Password: LDwwMK2y.
